# A Rare Case of Primary Squamous Cell Carcinoma of the Submandibular Salivary Gland: Brief Overview of Diagnostic Ambiguity and Treatment Challenges

**DOI:** 10.7759/cureus.28854

**Published:** 2022-09-06

**Authors:** Pawan Hingnikar, Anendd Jadhav, Nitin D Bhola

**Affiliations:** 1 Oral and Maxillofacial Surgery, Sharad Pawar Dental College and Hospital, Datta Meghe Institute of Medical Sciences, Wardha, IND

**Keywords:** salivary gland, submandibular salivary gland, major salivary gland, primary squamous cell carcinoma, squamous cell carcinoma

## Abstract

Primary squamous cell carcinoma of the submandibular salivary gland is a rarity with obscure etiology and atypical presentation. The features include a progressively enlarging swelling in the lateral neck just below the mandible, which is rarely tender. Surgery is the mainstay of the treatment, and the role of adjuvant therapies is not defined. Precise diagnosis demands a step-by-step systematic approach to exclude the presence of any index tumor. A 65-year-old countryside male presented to our institute with a complaint of submandibular swelling of the right side. After the exclusion of the primary, he was treated surgically with safe margins, ipsilateral comprehensive neck dissection, and adjuvant therapy. One month post-chemoradiotherapy, the patient developed a second primary in the contralateral submandibular region with lung metastasis and succumbed to death due to malignant cachexia. The stage of the disease at presentation, bone and skin involvement, lymphovascular invasion, poorer differentiation, and distant metastasis are associated with dismal outcomes. An early diagnosis and comprehensive surgical management with adjuvant chemoradiotherapy must be accomplished.

## Introduction

Primary squamous cell carcinoma (PSCC) of the major salivary gland (MSG) is a rare entity with obscure etiology and constitutes 1% to 4% of all head and neck cancers [1]. It most commonly involves the parotid gland, the submandibular salivary gland (SSG), and the sublingual gland in decreasing trend [2]. It is a unique, daunting disease with uncharacteristic presentation and often poses diagnostic and treatment challenges for the surgeon.

The diagnosis is established after exclusion of any history of radiotherapy and/or current evidence of PSCC of any other region of the head, neck, upper aerodigestive tract (UADT), and gastrointestinal tract (GIT). Given its rarity and uniqueness, comprehensive and definitive management is still a controversial topic. Many authors have attempted to review previous cases and solve the diagnostic and treatment challenges [3]. The present report aims to envisage the surgeon about this rarity and discuss its diagnostic ambiguity and management protocol.

## Case presentation

**Figure 1:**
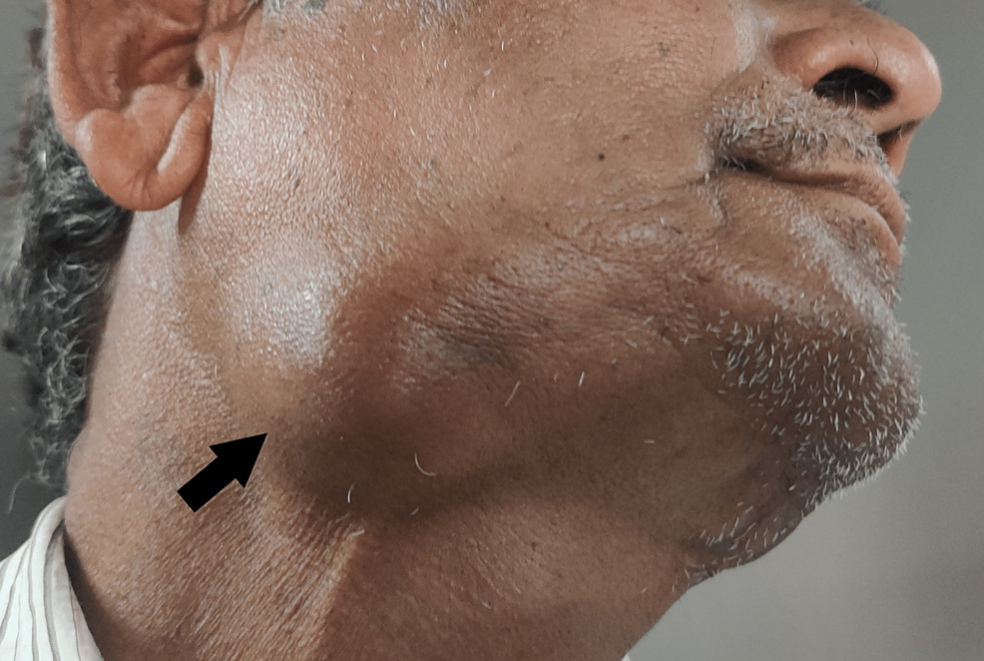
Clinical presentation of right submandibular region swelling.

A 65-year-old healthy, moderately built male presented with a complaint of painful, progressive swelling in the right submandibular region for four months (Figure 1). Clinical examination revealed a single, diffuse, tender swelling of 6x4 cm in its greatest dimension in the right submandibular region. The swelling was firm and fixed to the underlying structures and overlying skin. A primary clinical diagnosis of salivary gland tumor was considered with a differential diagnosis of metastatic disease or a PSCC of SSG. Contrast-enhanced computed tomography (CECT) revealed a 5.6 x 3.6 x 2.7 cm peripherally enhancing cystic lesion with a thick enhancing wall and septae within the right submandibular gland without bony involvement. To rule out the primary head, neck, and UADT lesion, a detailed clinical examination followed by pan endoscopy was performed with unremarkable findings.

Fine needle aspiration cytology (FNAC) from the swelling was performed, which showed deposits of moderately differentiated squamous cell carcinoma (SCC).

**Figure 2:**
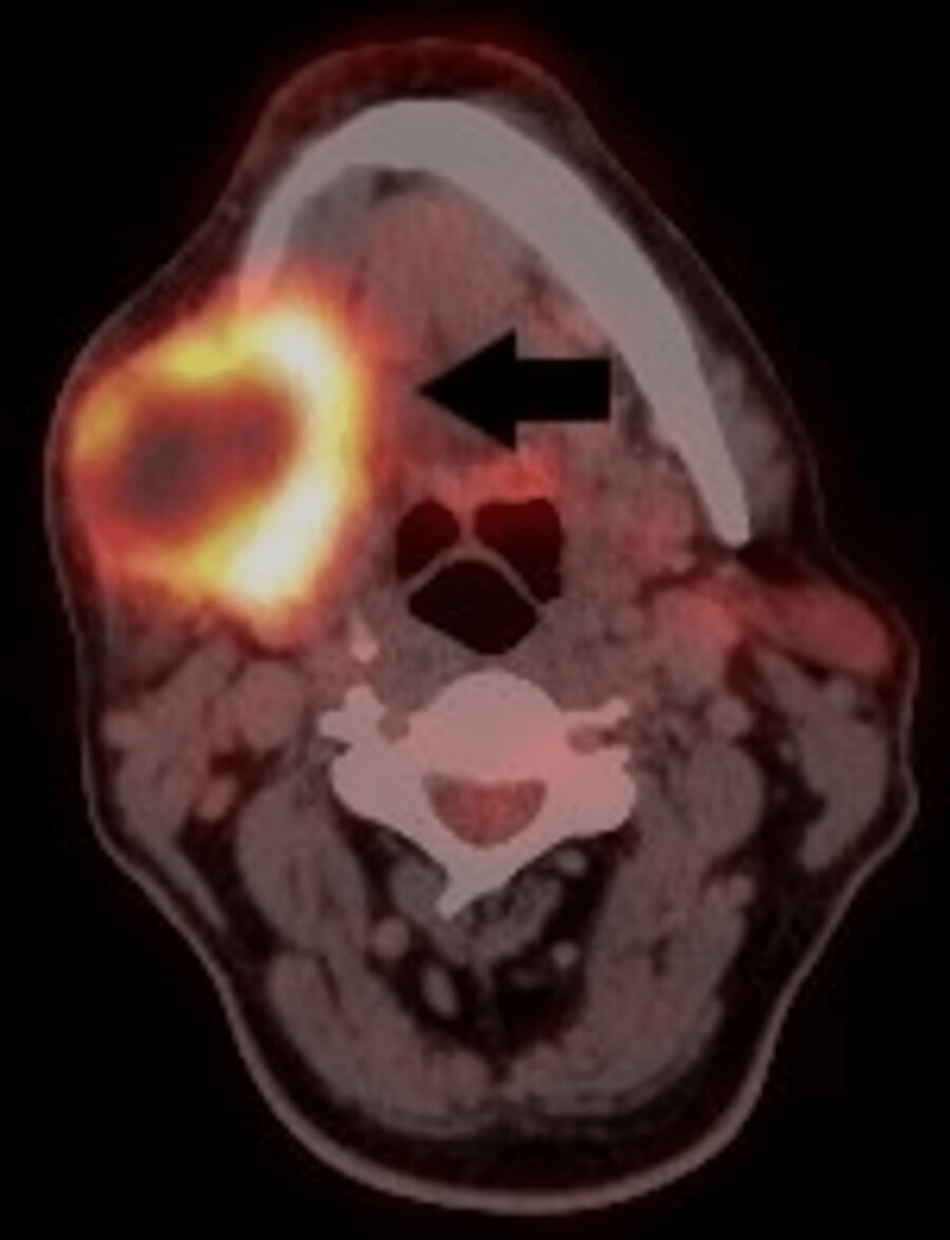
An 18F-FDG PET CT scan showing involvement of the submandibular salivary gland.

A whole-body 18F-FDG PET CT (18-fluorine-fluorodeoxyglucose positron emission tomography computed tomography) scan demonstrated increased FDG uptake in a large heterogeneously enhancing necrotic soft tissue density lesion (SUV max 13.3) in the right submandibular region measuring 5.5 x 4 cm without any distant spread (Figure 2). Based on clinical, imaging, and cytopathological characteristics, the final diagnosis of PSCC of SSG was made.

The patient was surgically treated by composite resection of tumor along with modified radical neck dissection (MRND) of the right side, and reconstruction with pectoralis major myocutaneous flap was performed.

**Figure 3:**
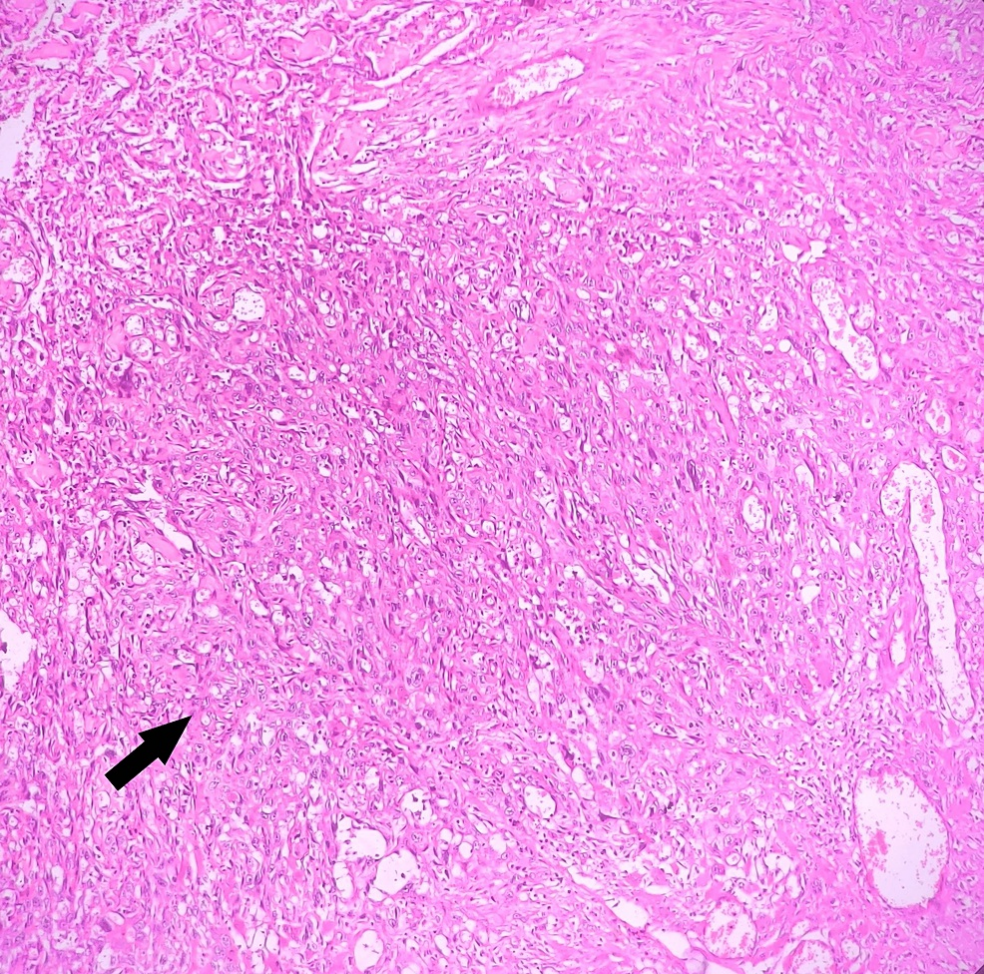
H&E stain showing malignant squamous cells.

Histopathological examination revealed sheets of neoplastic epithelial cells invading the connective tissue stroma in the form of dissociated cells and exhibiting features such as loss of cell-to-cell adhesion and cellular pleomorphism with vascular invasion. The connective tissue stroma showed severe chronic inflammatory cell infiltration chiefly comprising of lymphocytes with many endothelial lined blood vessels with intra- and extravasated red blood cells and haphazardly arranged cell fibers suggestive of poorly differentiated SCC (Figure 3). Metastatic deposits of tumor cells to pre- and post-vascular lymph nodes were also evident. A single level II lymph node was positive for metastatic deposits.

Postoperative recovery was uneventful. The patient was subjected to concurrent chemoradiotherapy (CCRT). One month post-CCRT, the patient developed a swelling in the contralateral submandibular region. FNAC from the swelling suggested deposits of SCC. A follow-up 18F-FDG PET CT scan of the whole body illustrated significant lung metastasis, and the patient succumbed to death due to malignant cachexia.

## Discussion

Commonly, the metastatic involvement of SSG occurs secondary to PSCC arising from the head, neck, and UADT. However, SCC of SSG without any index tumor of the head, neck, and UADT is a rarity. The characteristic presentation of PSCC of SSG is an asymptomatic progressive swelling, rarely tender, often fixed in the submandibular region. It most commonly occurs in the sixth decade of life with a distinct male predilection [4]. Our case carries a similar presentation of the disease.

Although obscure, a plausible etiology may be attributed to including the squamous epithelium during the SMG embryogenesis and subsequent dysplastic transformation. Once the diagnosis is ascertained, every attempt should be directed toward identifying the index tumor. Based on clinical and radiological examination, it is challenging to differentiate PSCC of MSG from other salivary gland malignancies. Histopathological examination is considered the gold standard to differentiate SCC from other tumors of MSG. Open biopsy of such lesions is a significant concern. However, FNAC appears to be the most appropriate pre-operative diagnostic modality, albeit with low sensitivity [5]. Immunohistochemistry (IHC) examination can differentiate between primary MSG lesions and metastatic cell deposits from the distant primary [6].

The challenges in pursuit of finding the primary tumor may be attributed to the small size of the lesion and location in difficult-to-access areas that can be frequently missed by routine clinical examination and imaging studies. A step-wise systematic approach should be adopted in the evaluation of such cases. CECT evaluation is the preferred first-line imaging study complemented with a thorough clinical examination and history. CECT evaluation should always precede pan-endoscopic examinations (UADT and GIT) and biopsy to facilitate precise diagnosis and sampling procedure. PET and PET CT have the utility in diagnosing the occult primary tumor. Our diagnostic approach toward the present case was concurrent with the literature recommendation.

The optimal regional treatment recommendations for primary SCC SMG are still a topic of debate in light of its rarity and lack of clinical studies. The literature survey recommends surgical resection of the lesion with safe surgical margins and comprehensive neck dissection with adjuvant therapies [7]. A reverse wedge mandibulectomy should be considered when the involved gland has caused pressure resorption, albeit a segmental resection is recommended in the presence of bone invasion. The age, gender, stage of the disease at presentation, bone and skin involvement, vascular invasion, nodal metastasis, and poorer differentiation are considered negative prognostic indicators.

Out of all the reported cases, many patients presented with either an early secondary pulmonary metastasis or recurrence of the lesion with dismal outcomes [3,4,6,8-14]. The reported survival rate of the patients with PSCC of MSG is 50% to 80% for low-grade tumors and around 14% for high-grade tumors [15].

Considering the aggressive tumor behavior, evidence of lymph node metastasis, and presence of vascular invasion, and anticipating a high risk of distant metastasis, our patient was treated with CCRT.

## Conclusions

To conclude, PSCC of MSG is a rarity with a distinct clinical entity, aggressive nature, and a high risk of distant metastasis, and necessitates an early diagnosis and prompt comprehensive surgical management and adjuvant CCRT. Considering the evidence from the literature and our experience with this rare entity, we recommend a comprehensive neck dissection, including MRND of both sides of the neck if contralateral nodes are also present, with adjuvant chemotherapy and radiotherapy for optimal survival and to avoid dismal outcomes.
